# Supplementary material for: Altered growth conditions more than reforestation counteracted forest biomass carbon emissions 1990–2020
Source: Nat Commun. 2021 Oct 19;12:6075. doi: 10.1038/s41467-021-26398-2 (PMC8526671; doi:10.1038/s41467-021-26398-2)
Supplement: Supplementary file 1 — Supllementary Information 1 [file 41467_2021_26398_MOESM1_ESM.pdf]

**Supplementary Information (SI1) to the paper:**

# Altered growth conditions – more than reforestation – counteracted forest biomass carbon emissions 1990-2020

Julia Le Noë[1a*], Karl-Heinz Erb[1], Sarah Matej[1], Andreas Magerl[1] Manan Bhan[1], Simone Gingrich[1]

[1]Institute of Social Ecology (SEC), Department of Economics and Social Sciences, University of Natural Resources and Life Sciences, Wien, Austria
[a]Current affiliation: Geology Laboratory, École Normale Supérieur, PSL University, Paris, France
*Corresponding author:
Julia Le Noë, julia.lenoe@boku.ac.at

*This SI provides additional figures to the main manuscript and results from the sensitivity analysis (Supplementary Note 1: figures and discussion) as well as additional information on the data and methodological approach (Supplementary Note 2: Supplementary methods) used to develop the CRAFT model at the national level for 152 countries of the world from 1990 to 2020.*

# Supplementary Note 1: Figures and discussion

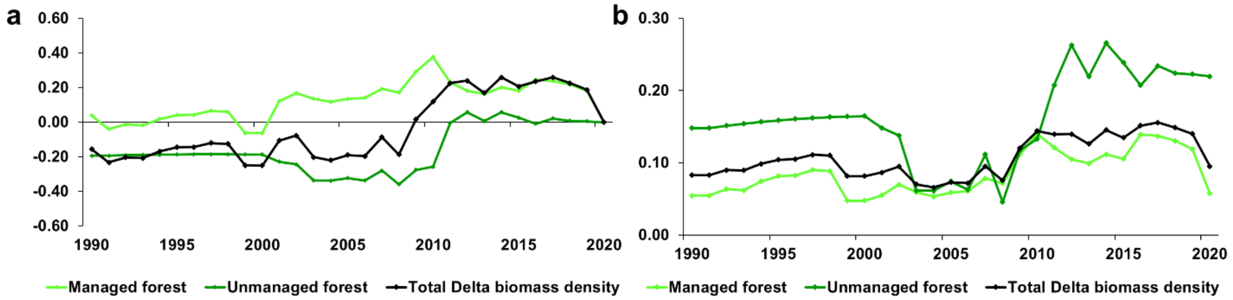


**Supplementary Figure 1.** Global trends in total, primary and managed forests (**a**) annual C net emissions (GtC) and; (**b**) annual net change in forest biomass C stock densities (tC/ha).

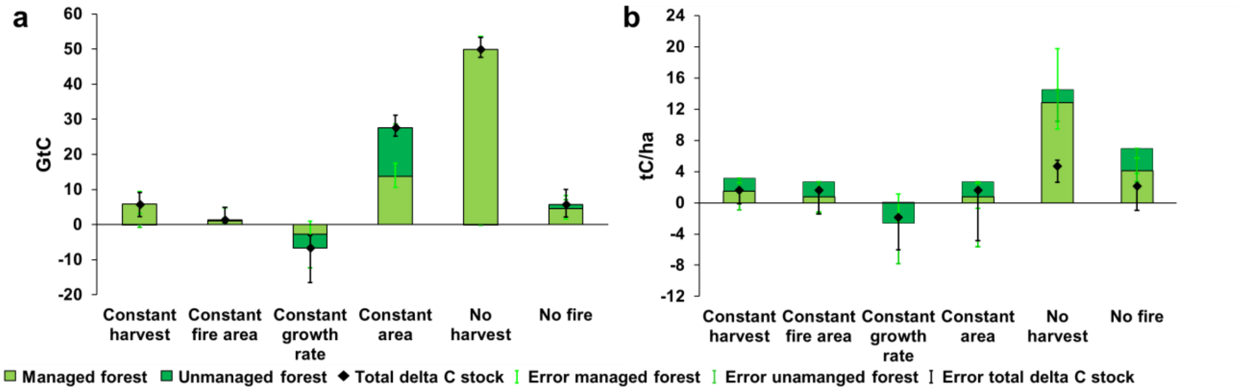


**Supplementary Figure 2.** Counterfactual scenarios (1990-2020) assessing the cumulative impact of: changes in harvest (**CF1**); changes in forest growth rate (**CF2**); changes in burnt area (**CF3**); changes in forest area (**CF4**); total harvest (**CF5**) and; total fire (**CF6**) on (**a**) C budget (GtC); (**b**) changes in forest biomass density (tC/ha), aggregated at the global level. Panels a and b show the difference between CF and actual C budgets and changes in biomass density respectively, with positive values indicating that the absence of the specific driver would result in higher C stocks.

As changes in area and growth are the two main drivers of trends in biomass C stocks, we perform a deeper analysis of the combined effects of these two drivers at the country level in 1990-2020. This analytical approach reveals four exclusive sectors of the combined effects of area and growth changes (Supplementary Figure 3). Area changes have a C source effect in 97 countries (sector I and III) but this effect is counteracted by growth changes contributing to a sink effect in 66 countries (sector III). The concomitant changes in growth and area foster a sink effect in 31 additional countries (sector IV) while afforestation alone contribute to a sink effect in only 24 countries (sector II). This again confirms that enhanced growth, more than reforestation, is the main driver counteracting biomass C emissions globally in 1990-2020.

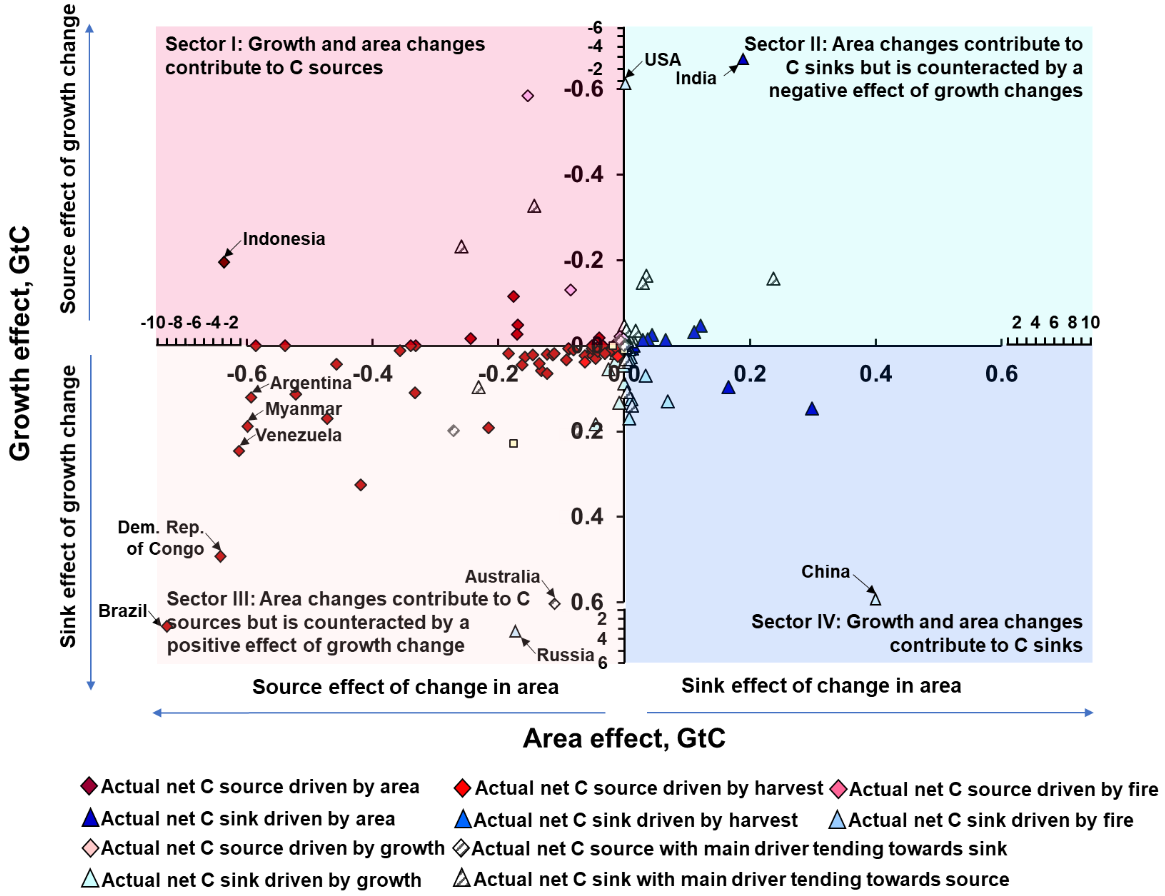


**Supplementary Figure 3**. Additional net C emissions with regards to actual net C emissions in the absence of change in forest area (x-axis; **CF4**) plotted against net C emissions with regards to actual net C emissions in the absence of change in forest growth (y-axis; **CF2**). Each dot represents a country with negative values indicating a C source effects of changes in area/growth and positive values indicating a C source effect of changes in area/growth. The graph shows four exclusive sectors according to the combination of sink or source effects of changes in area and growth. All dots are located within a range of values from -0.6 to 0.6 GtC, with the exceptions of Argentina (-0.76; -0.11), Australia (-0.61; 0.29), Brazil (-9.60; 2.97), China (0.44; -0.77), Democratic Republic of Congo (-3.72; 0.51), India (0.18; -3.09), Indonesia (-3.27; 0.20), Myanmar (-0.95; 0.17), Russia (-0.21; 3.14) and, the USA (0.01; -0.71).
.

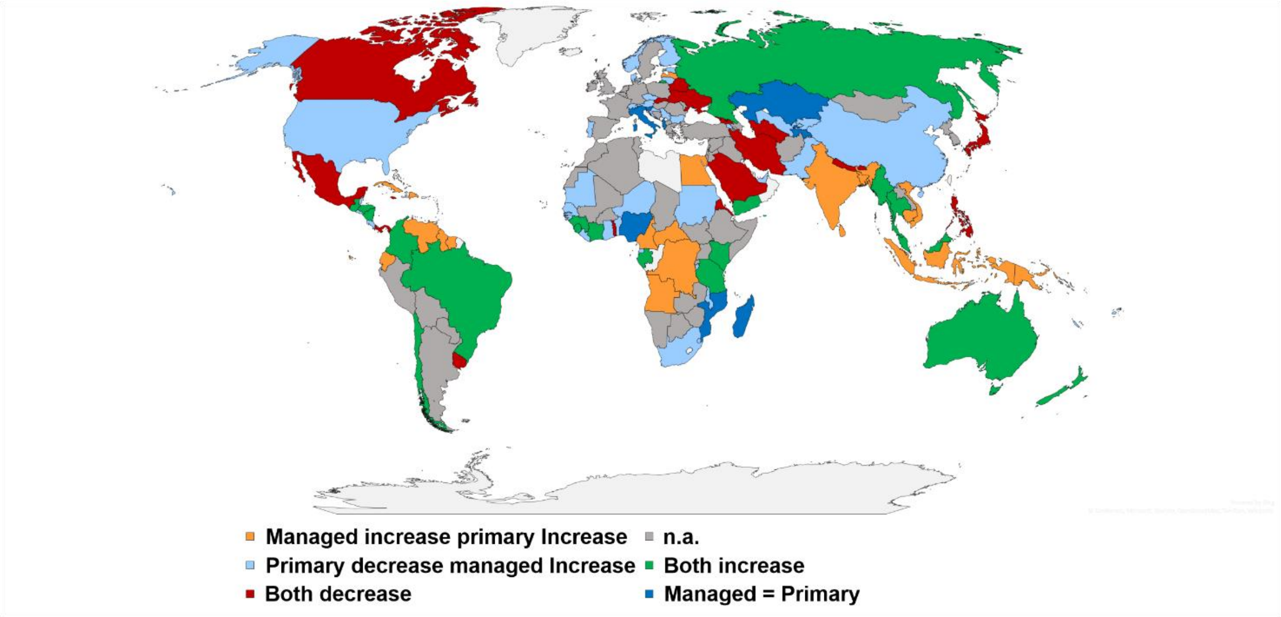


**Supplementary Figure 4.** Trends in growth rate change in primary and managed forests.

In order to assess the FRA datasets and model estimates reliability, we carried out a literature survey to provide independent comparisons between our modelled estimates/FRA dataset of forest biomass carbon stock changes and estimates by other independent studies (Supplementary Table 1). These comparisons reveal that our estimations are in good agreement with all other independent studies.

In addition, in order to assess the robustness of our modelling approach and results, we carried out 5 sensitivity analyses on the most uncertain parameters/assumptions: (i) temporal change in K parameter assumption and; (ii) changes in primary forest biomass density assumption; (iii) high fire impact assumption; (iv) low fire impact assumption; (v) effect of gross versus net area changes. Consequently, to assess the uncertainties associated to those assumptions, we set up these 5 sensitivity analyses as:

1- We tested (i) an alternative hypothesis in which both the temporal trend of the r and K parameters were optimized against the default assumption based on the optimization of the temporal change of the r parameter only.
2- We tested (ii) an alternative hypothesis in which biomass density in primary forest was taken as constant (using the provided in 2000 by Erb et al.[2]) against the default assumption based on an estimation of changes in primary forest biomass density (see Eq.4-7 in method section).
3- We tested (iii) an alternative hypothesis in which fire losses were calculated by assuming the highest fire severity coefficient (see Supplementary Tables 3 and 4) and without the best-guess correction of burnt area (assuming there was no overlap between forest area loss and burnt area).
4- We tested (iv) an alternative hypothesis in which fire losses were calculated by assuming the lowest fire severity coefficient (see Supplementary Tables 3 and 4) and by applying the best-guess correction of burnt area to all countries (and not only tropical countries as in the reference estimation).

5- We tested (v) an alternative hypothesis in which annual gross area gain and loss were estimated based on national conversion factor of gross to net area change derived from Li et al.[3] (see method section).

The effect of each sensitivity analysis on (i) the model performances, parameter optimization, global C budget estimates, (ii) counterfactual assessment of the forest biomass C-dynamic drivers and, (iii) the main figures of the results are summarized in Supplementary Tables 2 and3 and Supplementary Figures 5-10, respectively.

**Supplementary Table 1.** Comparison between forest biomass C budget as estimated by the CRAFT model, reported by the FRA dataset and estimated in other independent studies.

| Country or region | Period of time | Estimation method | Forest biomass C sink (Mt C $yr^{-1}$) | Source |
|---|---|---|---|---|
| Russia | 1990-2007 | Inventory data and field observations coupled to statistical or process models | 120 | Pan et al.[4] |
| | 1990-2007 | Modelling | 139 | Present Study |
| | 1990-2010 | Inventory | 164 | FRA[5] |
| Canada | 1990-2007 | Inventory data and field observations coupled to statistical or process models | -18 | Pan et al.[4] |
| | 1990-2007 | Modelling | -42 | Present Study |
| | 1990-2010 | Inventory | -44.7 | FRA[5] |
| USA | 1990-2007 | Inventory data and field observations coupled to statistical or process models | 129 | Pan et al.[4] |
| | 1990-2007 | Modelling | 121 | Present Study |
| | 1990-2010 | Inventory | 123 | FRA[5] |
| | 1992-2012 | National Inventory | 182 | Magerl et al.[6] |
| | 1992-2012 | Modelling | 144 | Present Study |
| | 1990-2010 | Inventory | 123 | FRA[5] |
| Europe | 1990-2007 | Inventory data and field observations coupled to statistical or process models | 125 | Pan et al.[4] |
| | 1990-2007 | Modelling | 126 | Present Study |
| | 1990-2010 | Inventory | 125 | FRA[5] |
| France | 1998-2011 | National inventory | 11 | IGN[7] |
| | 1998-2011 | Modelling | 14.7 | Present study |
| | 2000-2010 | Inventory | 19.6 | FRA[5] |
| Austria | 1994-2017 | National inventory | 3.5 | BFW[8] |
| | 1994-2017 | Modelling | 2.2 | Present study |
| | 1990-2017 | Inventory | 2.5 | FRA[5] |
| China | 1990-2007 | Inventory data and field observations coupled to statistical or process models | 83 | Pan et al.[4] |
| | 1990-2007 | Modelling | 97.7 | Present Study |
| | 1990-2010 | Inventory | 93.4 | FRA[5] |

| | | | | |
|---|---|---|---|---|
| | 1990-2000 | National Inventory | 75 | Fang et al.[9] |
| | 1990-2000 | Modelling | 68.5 | Present Study |
| | 1990-2000 | Inventory | 95.7 | FRA[5] |
| Japan | 1990-2007 | Inventory data and field observations coupled to statistical or process models | 24 | Pan et al.[4] |
| | 1990-2000 | Modelling | 24.6 | Present Study |
| | 1990-2010 | Inventory | 25.0 | FRA[5] |
| | 1990-2000 | National Inventory | 22 | Fang et al.[9] |
| | | Modelling | 23.6 | Present Study |
| | | Inventory | 22.0 | FRA[5] |
| South Korea | 1990-2007 | Inventory data and field observations coupled to statistical or process models | 8.4 | Pan et al.[4] |
| | 1990-2007 | Modelling | 13.2 | Present Study |
| | 1990-2010 | Inventory | 14.0 | FRA[5] |
| | 1990-2000 | National Inventory | 8.3 | Fang et al.[9] |
| | 1990-2000 | Modelling | 11.5 | Present Study |
| | 1990-2000 | Inventory | 11.6 | FRA[5] |
| India | 1994-2010 | National Inventory | 28.5 | Rajashekar et al.[10] |
| | 1994-2010 | Modelling | 30.0 | Present Study |
| | 1990-2010 | Inventory | 31.2 | FRA[5] |
| Global tropic | 1990-2007 | Inventory data and field observations coupled to statistical or process models | -478 | Calculated from Pan et al.[4] |
| | 1990-2007 | Modelling | -646 | Present Study |
| | 1990-2010 | Inventory | -685 | FRA[5] |
| | 2003-2014 | Satellite data | -425 | Baccini et al.[11] |
| | 2003-2014 | Modelling | -534 | Present Study |
| | 2000-2015 | Inventory | -637 | FRA[5] |
| | 1990-2010 | Satellite data combined with statistical model | -778 | Achard et al.[12] |
| | 1990-2010 | Modelling | -647 | Present study |
| | 1990-2010 | Inventory | -685 | FRA[5] |
| Congo | 1990-2017 | Lidar remote sensing coupled with regional inventory report | -114 | Combining change in forest area (FRA) and biomass density reported by Xu et al.[13] |
| | 1990-2017 | Modelling | -116 | Present study |
| | 1990-2017 | Inventory | -117 | FRA[5] |

**Supplementary Table 2.** Estimates of global weighted average of r (tC $tC^{-1}$), K (tC $ha^{-1}$) and α (% $an^{-1}$) parameters, global weighted average of relative root means square error (RMSE, %), and global C budgets (GtC) in 1990-2020 by the reference model assumptions, by the five sensitivity analyses and by the FRA. Negative C budget indicates net C emissions from forest biomass.

| | Dynamic K parameter | Constant primary forest biomass density | High fire estimates | Low fire estimates | Gross area change | Reference assumptions | FRA |
|---|---|---|---|---|---|---|---|
| r parameter primary | 0.09 | 0.09 | 0.09 | 0.07 | 0.14 | 0.10 | X |
| K parameter primary | 252 | 251 | 250 | 249 | 266 | 243 | X |
| α parameter primary | 0.17% | 0.17% | 0.17% | 0.19% | 0.19% | 0.19% | X |
| RMSE primary | 1.39% | 0.45% | 0.73% | 0.62% | 0.86% | 0.45% | X |
| r parameter managed | 0.07 | 0.07 | 0.07 | 0.06 | 0.12 | 0.07 | X |
| K parameter managed | 313 | 313 | 315 | 309 | 321 | 313 | X |
| α parameter managed | 0.21% | 0.21% | 0.21% | 0.21% | 0.23% | 0.21% | X |
| RMSE managed | 1.41% | 0.62% | 1.06% | 1.05% | 2.39% | 0.62% | X |
| r parameter total | 0.09 | 0.09 | 0.09 | 0.09 | 0.14 | 0.09 | X |
| K parameter total | 269 | 262 | 270 | 262 | 280 | 262 | X |
| α parameter total | 0.20% | 0.20% | 0.20% | 0.20% | 0.22% | 0.21% | X |
| RMSE total | 1.41% | 0.57% | 0.95% | 0.93% | 1.94% | 0.57% | X |
| Primary C budget | 0.70 | -4.68 | -6.01 | -4.00 | -4.42 | -4.68 | -4.74* |
| Managed C budget | 7.74 | 4.02 | 0.85 | 6.20 | 0.22 | 3.94 | 3.05* |
| Total global C budget | 8.44 | -0.66 | -5.16 | 2.25 | -4.19 | -0.74 | -1.69* |

*C budgets are derived from the FRA datasets by applying Eq.4-7 (see method section).

**Supplementary Table 3.** Ranking of the divergences between the actual and counterfactual assessments of global C budgets as calculated in the five sensitivity analyses *versus* by the reference model simulation. Number 1 and 5 respectively denote the maximum and minimum divergences with the reference simulation. Figures in parenthesis provide global C budgets in GtC in 1990-2020 for actual and counterfactual assessments. Negative C budget indicates net C emissions from forest biomass.

| | Dynamic K parameter | Constant primary forest biomass density | High fire estimates | Low fire estimates | Gross area change | Reference assumptions |
|---|---|---|---|---|---|---|
| Actual C budget | 1 (9.26 GtC) | 5 (-0.66 GtC) | 2 (-5.16 GtC) | 4 (2.25 GtC) | 3 (-4.19 GtC) | (-0.74 GtC) |
| CF1 C budget (no harvest changes) | 1 (14.4 GtC) | 4 (4.75 GtC) | 2 (0.22 GtC) | 5 (4.92 GtC) | 3 (0.72 GtC) | (4.92 GtC) |
| CF2 C budget (no growth rate changes) | 5 (-7.61 GtC) | 5 (-7.61 GtC) | 2 (-12.1 GtC) | 3 (-5.32 GtC) | 1 (-13.1 GtC) | (-7.45 GtC) |
| CF3 C budget (no fire changes) | 1 (10.2 GtC) | 5 (0.46 GtC) | 3 (-3.23 GtC) | 4 (3.14 GtC) | 2 (-3.27 GtC) | (0.63 GtC) |
| CF4 C budget (no area changes) | 1 (37.5 GtC) | 5 (26.6 GtC) | 3 (21.7 GtC) | 4 (30.4 GtC) | 2 (20.8 GtC) | (26.9 GtC) |
| CF5 C budget (no harvest) | 1 (57.6 GtC) | 5 (48.9 GtC) | 3 (44.4 GtC) | 4 (52.3 GtC) | 2 (43.1 GtC) | (49.1 GtC) |
| CF6 C budget (no fire) | 1 (14.6 GtC) | 5 (4.74 GtC) | 5 (4.74 GtC) | 5 (4.74 GtC) | 2 (-0.32 GtC) | (5.40 GtC) |

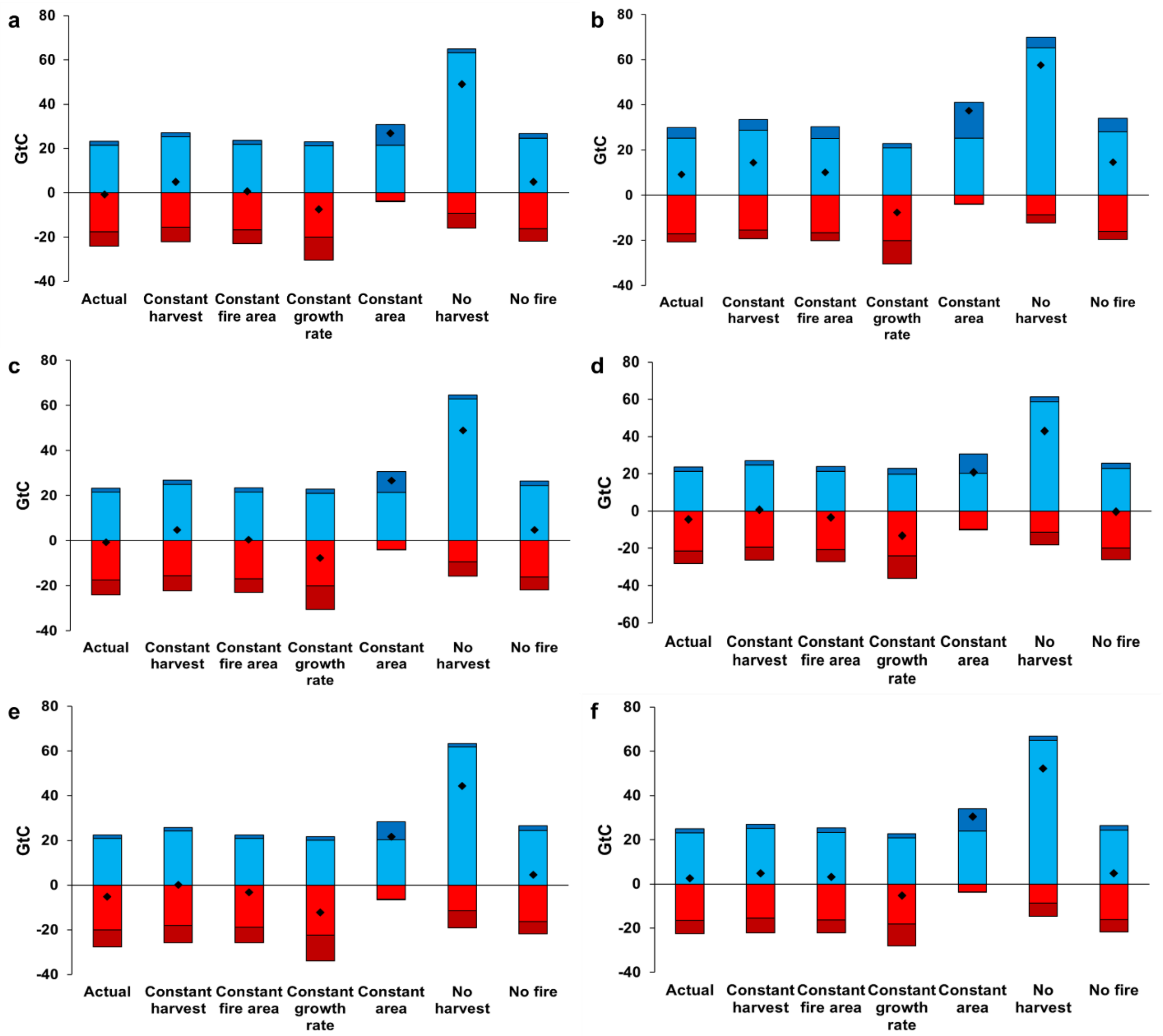


**Supplementary Figure 5.** Sensitivity analysis of the counterfactual scenarios (1990-2020) assessing the cumulative impact of: change in harvest (CF1); change in forest growth rate (CF2); change in burnt area (CF3); change in forest area (CF4); total harvest (CF5) and; total fire (CF6) on C budget (GtC) with (**a**) reference model; (**b**) dynamic K assumption; (**c**) constant primary biomass density assumption; (**d**) estimates of gross area changes; (**e**) high fire estimates; (**f**) low fire estimates.

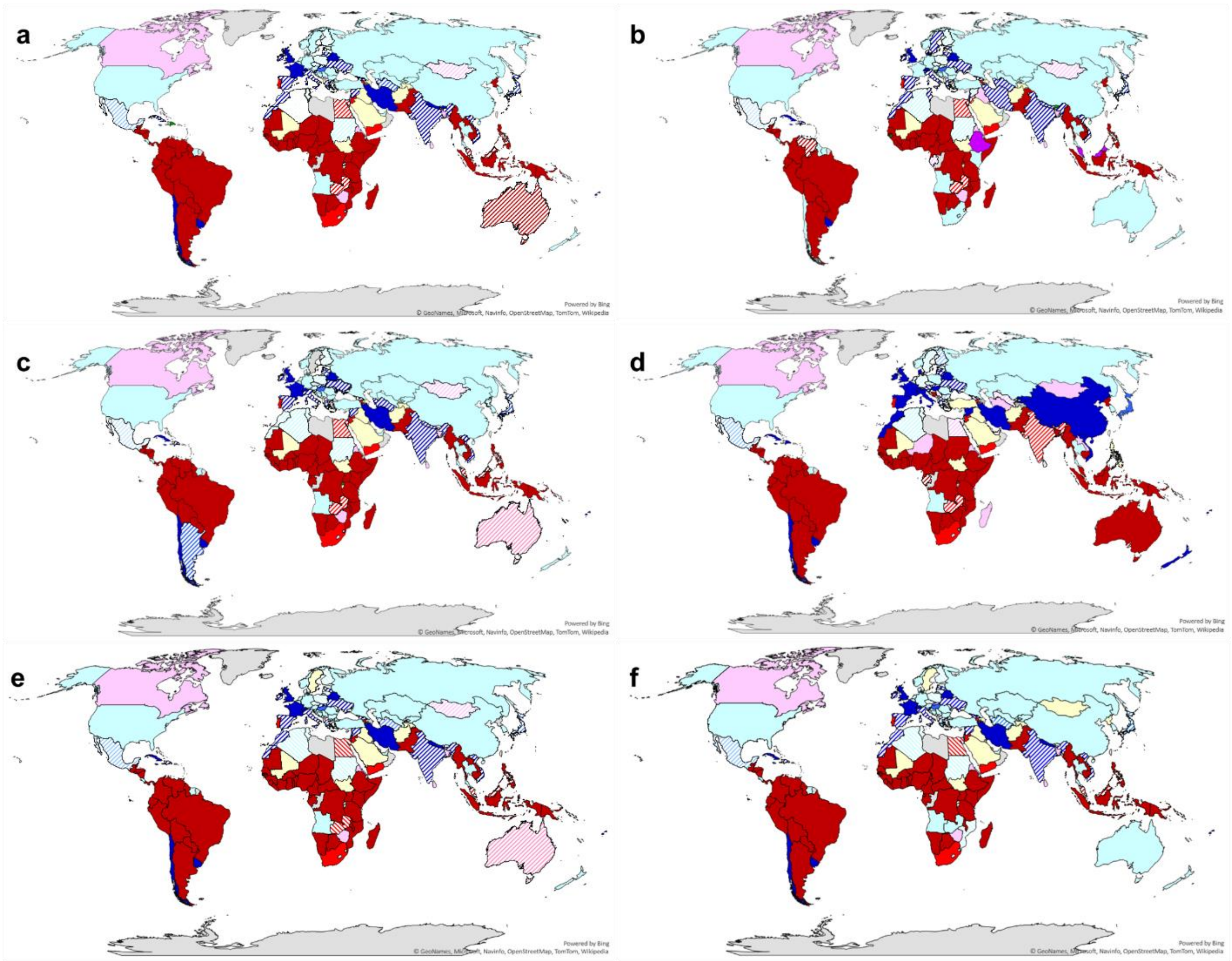


**Supplementary Figure 6.** Sensitivity analysis of the main drivers of the net C emissions from forest biomass at the national level in 1990-2020 according to the Boolean typology using the results from the counterfactual scenario assessment as criteria (see Fig. 3b in the main manuscript) with (**a**) reference model; (**b**) dynamic K assumption; (**c**) constant primary biomass density assumption; (**d**) estimates of gross area changes; (**e**) high fire estimates; (**f**) low fire estimates. The hatches stand for cases in which the driver with the strongest effect counteract the observed C budget. The colour of the hatches corresponds to the main factor identified by the decision tree algorithm.

China switches from a net C sink driven by increase growth rate to a net C sink driven by reforestation in the gross area change sensitivity analysis (Supplementary Figure 6d). This is due to the fact that both area and growth rate changes have positive effects on the net C sink in China (Supplementary Figure 3) so that changes in model assumption could result in further highlighting one driver rather than the other.

India switches from a net C source driven by afforestation to a net C source driven by increased harvest pressure in the gross area change sensitivity analysis (Supplementary Figure 6d). Considering gross area changes in India resulted in such a decrease of biomass density (due to increase rejuvenation through higher reforested area) that harvest pressure became higher that annual NPP. In such conditions, the CRAFT model could not reproduce the FRA data in Indian managed forest, which even collapsed by the end of the simulation in this sensitivity analysis, thus suggesting that the average gross-net area change ratio of c.12 in India[3] might be unrealistic.

Australia switches from a net C sink driven by deforestation to a net C sink driven by increased growth rate in the dynamic K and low fire sensitivity analyses (Supplementary Figures 6b and f) but

also to a net C source driven by increased fire intensity in the constant primary biomass and high fire sensitivity analyses (Supplementary Figures 6c and e). These changes in the type of C-dynamics trajectory identified in Australia are due to the fact that several drivers had large but contradicting effects in these countries: Both increased fire intensity and deforestation contributed to a net C source while increased growth rate was actually the largest driver counteracting the C source in the reference model simulation (Supplementary Figure 3). In those conditions, it is unsurprising that changes in the model assumption and parameters are more likely to affect the C budget and main driver attribution in this country.

In addition, it is worth noting that many West European countries switch from a net C sink driven by increased growth rate to a net C sink driven by reforestation and *vice-versa* across the different sensitivity analyses. These changes result from the synergetic effects of both reforestation and growth rate changes in these countries (Supplementary Figure 3), so that changes in model assumption may result in further highlighting one driver rather than the other.

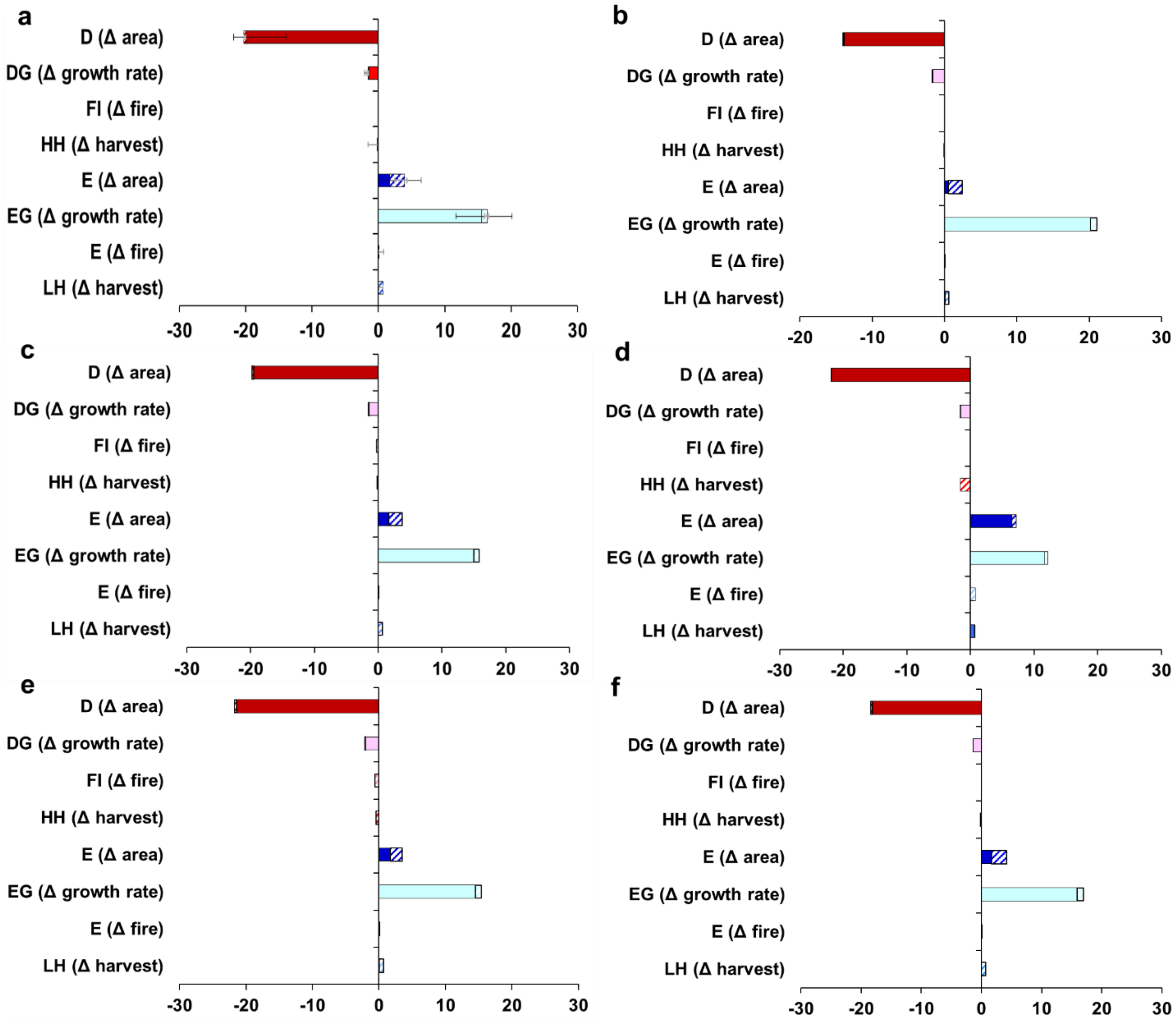


**Supplementary Figure 7.** Sensitivity analysis of the sum of net C sinks and net C sources in each type of forest C-dynamics trajectory identified through the typology (see Fig. 3b in the main manuscript) with (**a**) reference model; (**b**) dynamic K assumption; (**c**) constant primary biomass density assumption; (**d**) estimates of gross area changes; (**e**)

high fire estimates; (**f**) low fire estimates. The hatches stand for cases in which the driver with the strongest effect evaluated by the counterfactual scenarios counteract the observed carbon budget. The colour of the hatches corresponds to the main factor identified by the decision tree algorithm.

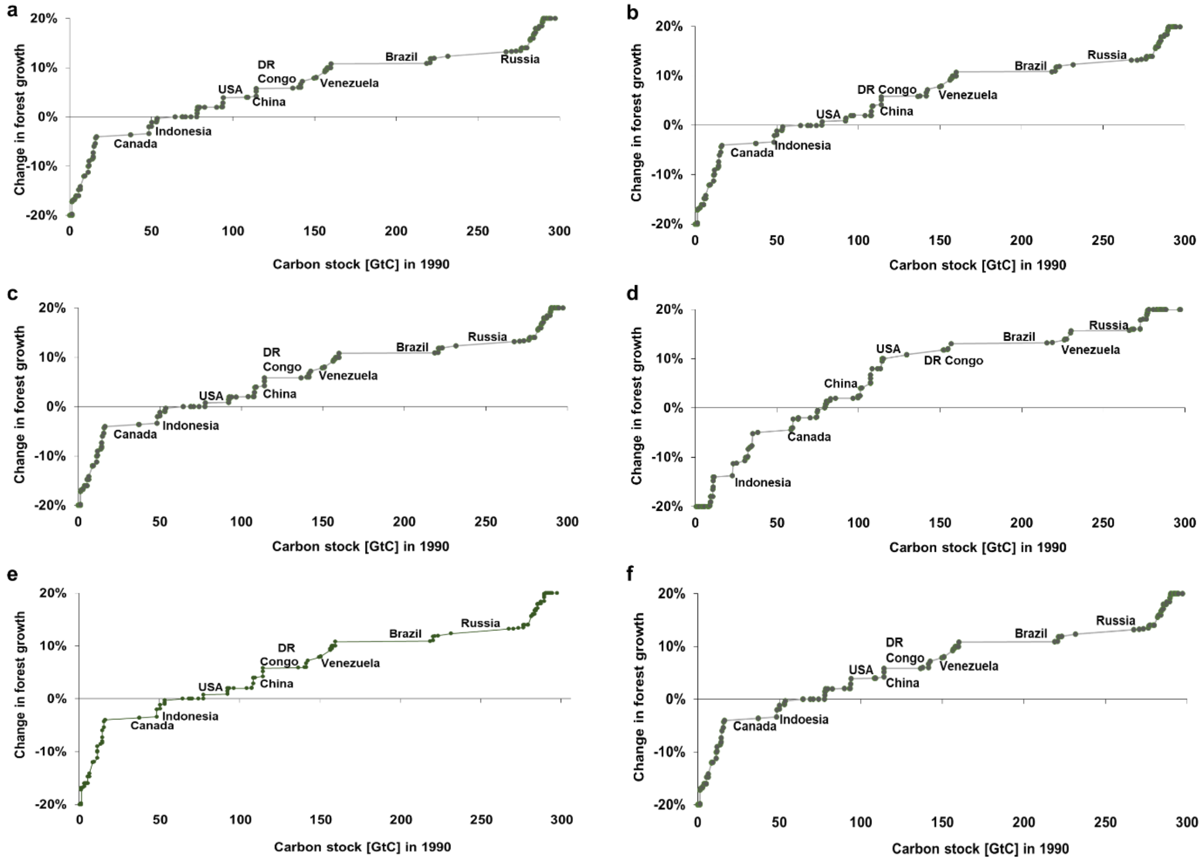


**Supplementary Figure 8.** Sensitivity analysis of change in forest growth rate and its effects on global carbon stocks in **total forest** for (**a**) reference model; (**b**) dynamic K assumption; (**c**) constant primary biomass density assumption; (**d**) estimates of gross area changes; (**e**) high fire estimates; (**f**) low fire estimates. The diagrams show national forest growth rate changes (y-axis) scaled along the cumulated size of the carbon stock in 1990 (x-axis). The area between the graph and the x-axis indicates the C-stock change due to growth rate.

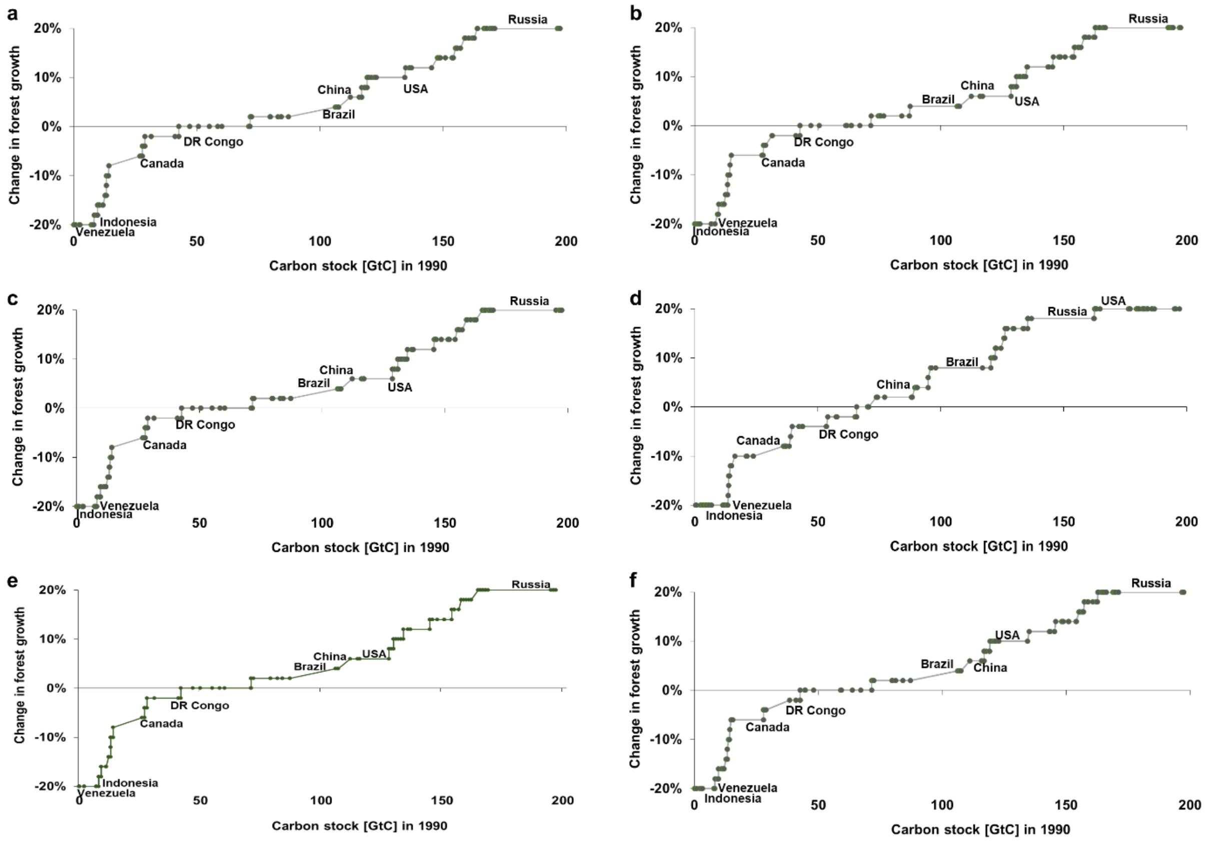


**Supplementary Figure 9.** Sensitivity analysis of change in forest growth rate and its effects on global carbon stocks in **managed forest** for (**a**) reference model; (**b**) dynamic K assumption; (**c**) constant primary biomass density assumption; (**d**) estimates of gross area changes; (**e**) high fire estimates; (**f**) low fire estimates. The diagrams show national forest growth rate changes (y-axis) scaled along the cumulated size of the carbon stock in 1990 (x-axis). The area between the graph and the x-axis indicates the C-stock change due to growth rate.

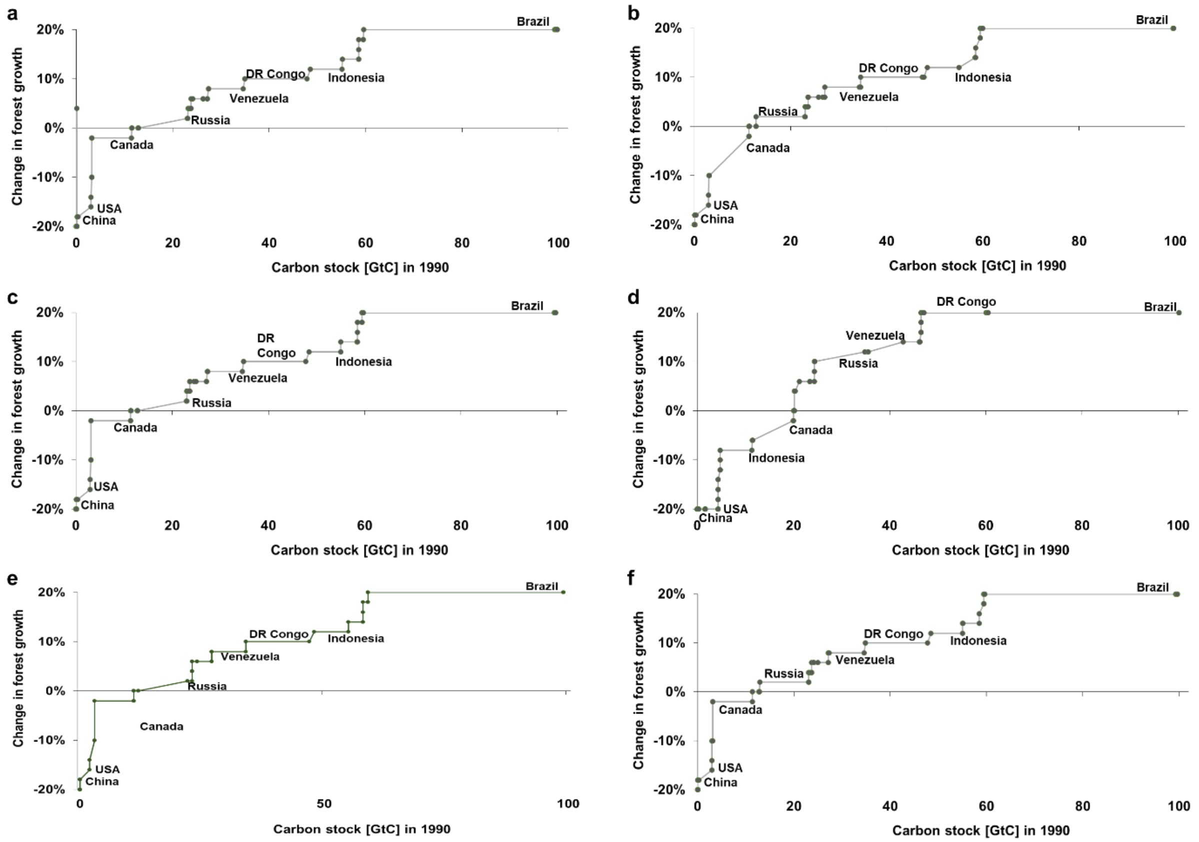


**Supplementary Figure 10.** Sensitivity analysis of change in forest growth rate and its effects on global carbon stocks in **primary forest** for (**a**) reference model; (**b**) dynamic K assumption; (**c**) constant primary biomass density assumption; (**d**) estimates of gross area changes; (**e**) high fire estimates; (**f**) low fire estimates. The diagrams show national forest growth rate changes (y-axis) scaled along the cumulated size of the carbon stock in 1990 (x-axis). The area between the graph and the x-axis indicates the C-stock change due to growth rate.

# Supplementary Note 2: methods

*This note is decomposed in 4 sections: 1- Additional explanation on the derivation of a range of values for the r and K parameters; 2- Additional information on the data used to estimated fire efficiency coefficient from a literature survey; 3- Additional information on the data used to derive coefficient of biomass density annual change from a literature survey. All references cited are provided at the end of the document.*

## 1. Deriving a range of possible values for the r and K parameters

In the present study, we derived a range of possible values for the growth rate parameters based on site measurement data on standing biomass and NPP. Site measurement data on standing biomass and NPP were compiled from Cannell[14], Luyssaert et al.[15] and Anderson-Teixeira et al.[16], contributing 372, 503 and 536 data points, respectively (Supplementary Table 4).

**Supplementary Table 4**. Summary of available data from Cannell[14], Luyssaert et al.[15] and Anderson-Teixeira et al.[16]. For the database from Luyssaert it was assumed that all measurements were taken from deciduous trees since only tropical sites were used.

| | | | | | | Standing biomass | | NPP | |
|---|---|---|---|---|---|---|---|---|---|
| [Number of data points] | N of site measure ments | Deci-duous | Conifer-ous | mixed | plantation | Abovegr ound (= woody structura l + foliage) | Total (abovegr ound + belowgr ound) | Abovegr ound (= woody structura l + foliage) | Total (abovegr ound + belowgr ound) |
| Cannell[14] | 372 | 278 | 88 | 6 | 24 | 371 | 177 | 224 | 74 |
| Luyssaert et al.[15] | 503 | 503 | 0 | 0 | 54 | 317 | 82 | 43 | 30 |
| Anderson-Teixeira et al.[16] | 536 | 230 | 304 | 2 | 39 | 498 | 530 | 475 | 457 |

The expansion factors, *i.e.*, the allometric coefficients between tree stems and other tree parts, per ecoregion given in Supplementary Table 5were used when data reported on biomass missed tree organs.

**Supplementary Table 5**. Used expansion factors derived as mean value of all site measurements in one ecoregion. Values in bracket are the mean over all ecoregions since no data for this ecoregion was available.

| **FAO ecoregions** | from aboveground standing biomass to total (above and belowground) | from stem NPP to total structural (woody) NPP | from structural (woody) NPP to total aboveground NPP | from structural (woody) NPP to total NPP (above and belowground) |
|---|---|---|---|---|
| tropical rainforest | 1.13 | 1.40 | 2.57 | 4.18 |
| tropical moist deciduous forest | 1.16 | 1.08 | 2.25 | 3.67 |
| tropical dry forest | 1.25 | (1.73) | 3.46 | 4.34 |
| tropical shrubland | 1.18 | (1.73) | (2.09) | (3.38) |
| tropical desert | (1.22) | (1.73) | (2.09) | (3.38) |
| tropical mountain system | 1.30 | 1.92 | 2.98 | 6.90 |
| subtropical humid forest | 1.26 | 1.28 | 2.24 | 4.18 |
| subtropical dry forest | 1.27 | 3.97 | 2.09 | 2.33 |
| subtropical steppe | (1.22) | 2.08 | 1.92 | 1.95 |
| subtropical desert | (1.22) | 2.14 | 1.99 | (3.38) |
| subtropical mountain system | 1.27 | 1.26 | 1.60 | 2.08 |
| temperate oceanic forest | 1.23 | 1.47 | 1.62 | 2.31 |
| temperate continental forest | 1.25 | 1.74 | 1.79 | 2.59 |
| temperate steppe | 1.25 | 1.85 | 1.29 | (3.38) |
| temperate desert | (1.22) | (1.73) | (2.09) | (3.38) |
| temperate mountain system | 1.25 | 1.24 | 1.70 | 2.57 |
| boreal coniferous forest | 1.20 | 1.35 | 1.61 | 2.92 |
| boreal tundra woodland | (1.22) | (1.73) | (2.09) | (3.38) |
| boreal mountain system | 1.22 | 1.40 | 1.77 | 4.61 |
| polar | 1.12 | (1.73) | 2.54 | 2.65 |

The following equation was fitted to best reproduce the site measurements in each ecoregion:

$$NPP=rB(1-B/K)$$

With r being the growth rate per year and K denoting the maximum carrying capacity (tC/ha). The r and K parameter of the curve with the smallest residues to the data points were identified (using the MATLAB function fmincon), under the constraints of r being between 0.01 and 1 and K being between 0 and 1000. The goodness of fit of the fitted function was also calculated. To arrive at a range of r and K values data was only selectively used for the fitting of the function:

- for the minimum values of r and K: per 0.2 percentile of Standing biomass values only NPP values below the median were used
- for the maximum values of r and K: per 0.2 percentile of Standing biomass values only NPP values above the median were used

From this approach, the lower deciles were 0.03 $yr^{-1}$ and 100 tC $ha^{-1}$ and the upper deciles were 0.21 $yr^{-1}$ and 720 tC $ha^{-1}$ respectively for the r and K values. In the present study, we used this range for all countries of the world to optimize the growth parameters at the country level.

# 2. Estimation of macro-regional fire efficiency coefficients

Studies estimating burning of biomass by wildfires usually apply region and ecosystem specific factors for fuel loads, subdivided into surface fuels (grasses, herbs, shrubs, litter, duff, coarse woody debris, roots), standing deadwood ("snags") and crown or canopy fuels (branches, twigs, foliage, bark), given in percentage or dry matter or C per area. These fuels represent the total potential combustible biomass per area burned, which varies widely across biomes, ecosystems and fuel compartments. Estimating the actual fraction of total biomass within an ecosystem that could burn in a fire event requires multiplying these fuels by respective combustion efficiency factors. We derived published fuel load factors (Supplementary Table 6), and combustion efficiencies for low, high, and best guess fire intensities (Supplementary Table 7) for forest biomes of the world from the literature and allocated them to the countries considered in this study. Since our aim was to model the losses of woody live biomass only, these tables represent fuel loadings and combustion efficiencies for wood (stem and branches) and foliage (leaves and needles).

**Supplementary Table 6.** Fuel loadings as percentage of total tree compartment for regions and respective biomes of the world, as used by Hoelzemann et al.[17]

| Region | Biome | Biome[1] | Foliage[1] (%) | Wood[1] |
|---|---|---|---|---|
| Southern Asia | boreal | Boreal forest, Eurasia | 20 | 20 |
| Central Asia and Russian Federation | boreal | Boreal forest, Eurasia | 20 | 20 |
| Northern America | boreal | Boreal forest, America | 10 | 30 |
| Western Europe | boreal | Boreal forest, Eurasia | 20 | 20 |
| Latin America & the Caribbean | boreal | Boreal forest, America | 10 | 30 |
| Southern Asia | temperate | Temperate forest | 30 | 10 |
| Eastern & South-Eastern Europe | temperate | Temperate forest | 30 | 10 |
| Northern Africa and Western Asia | temperate | Temperate forest | 30 | 10 |
| Western Europe | temperate | Temperate forest | 30 | 10 |
| Central Asia and Russian Federation | temperate | Temperate forest | 30 | 10 |
| Latin America & the Caribbean | temperate | Temperate forest | 30 | 10 |
| Oceania and Australia | temperate | Temperate forest | 30 | 10 |
| South-Eastern Asia | temperate | Temperate forest | 30 | 10 |
| Eastern Asia | temperate | Temperate forest | 30 | 10 |
| Sub-Saharan Africa | temperate | Temperate forest | 30 | 10 |
| Northern America | temperate | Temperate forest | 30 | 10 |
| Oceania and Australia | tropical | Tropical forest | 47 | 17 |
| Sub-Saharan Africa | tropical | Tropical forest | 47 | 17 |
| Latin America & the Caribbean | tropical | Tropical forest | 47 | 17 |
| Southern Asia | tropical | Tropical forest | 47 | 17 |
| South-Eastern Asia | tropical | Tropical forest | 47 | 17 |
| Eastern Asia | tropical | Tropical forest | 47 | 17 |
| Northern Africa and Western Asia | tropical | Tropical forest | 47 | 17 |
| America | tropical | Tropical forest | 47 | 17 |
| Africa | tropical | Tropical forest | 47 | 17 |
| Asia | tropical | Tropical forest | 47 | 17 |

**Supplementary Table 7.** Range of combustion efficiency factors per biome and fuel type as reported in Mouillot et al.[18]; Yang et al.[19]; Kloster et al.[20]; van Leeuwen et al.[21]

| | Combustion Efficiency per fuel type | | | | | |
|---|---|---|---|---|---|---|
| | Wood | | | Foliage | | |
| | Low | Best Guess | High | Low | Best Guess | High |
| Tropical | 0.10 | 0.30 | 0.50 | 0.30 | 0.60 | 0.9 |
| Temperate | 0.10 | 0.40 | 0.70 | 0.30 | 0.65 | 1.0 |
| Boreal | 0.10 | 0.40 | 0.70 | 0.30 | 0.65 | 1.0 |

Based on these coefficients (Supplementary Tables 6-7), we thus calculated three fire severity levels (low, moderate, high), hence assessing a range of coefficients ($fire\ severity\ (\%) = \sum_i C_i \times FL_i \times \alpha_i$), which were used to perform sensitivity analyses (see above). We used the moderate fire severity coefficients (ranging from 4 to 11% across world countries) to run the reference simulation (figures displayed in the main manuscript). These rather low coefficients are in contrast with other coefficients reported in the literature[21,22], however this is due to the fact that we only consider fire loss from living biomass while most C emissions from fire are from litter and dead wood[21–23].

# 3. Temporal trend in primary forest biomass density

## 3.1. General approach (Already presented in the method of the main manuscript)

In the present study we calculated the biomass C stocks of primary versus production forests by using the benchmark values of primary forest biomass density provided by Erb et al.[2] for the year 2000 at the country level. From this data, we could calculate the biomass C stocks in both primary and production forest in 2000 such as:

$$B_{prim\ 2000} = BD_{prim2000} \times A_{prim2000} \quad \text{[Eq. 1]}$$
$$B_{prod\ 2000} = B_{tot2000} - B_{prim2000} \quad \text{[Eq. 2]}$$

With $B_{prim2000}$, $B_{prod2000}$ and $B_{tot\ 2000}$ the C stocks biomass in 2000 respectively in primary, production and total forest. Before and after 2000 forest biomass density of production and primary forest may evolve following different trends, as empirical studies revealed that primary forests are not in equilibrium and may undergo changes in biomass density[24]. Indeed, primary forests all over the world are experiencing greening trends, i.e., increasing productivity and biomass densities, as well as browning trends, i.e., decreasing productivity and biomass densities. These greening and browning trends depends on a variety of drivers (see Supplementary Table 8).

**Supplementary Table 8.** Summary of the main factors driving greening & browning trends in primary forests

| | Direct climatic driver | Indirect climatic driver | Direct human-induced drivers |
|---|---|---|---|
| Greening trend | Longer growth period (temperature increase lengthens the period of growth in autumn and spring)<br>Higher summer peak in photosynthesis (also enabled by higher temperature) | $CO_2$ fertilization<br>Nitrogen deposition | Better legislation protection (limit primary forest loss and degradation) |
| Browning trend | Decreased vegetation due to temperature-induced drought stress | Increase infestations of insect pests (bark beetle) due to higher temperature favouring survival<br>Increase wild fire following increased temperature and dryness. | Logging (commercial concession)<br>Hunting<br>Land cover change |

In order to consider this pattern, we carried out a literature survey to derive temporal trends in biomass density of primary forests in boreal, temperate, paleotropical and neotropical forests. Subsequently, we used these macro-regional coefficients to derive biomass C stocks in production and primary forest at the national level over the 1990-2020 period from the benchmark values of 2000 using the following relationships:

$$B_{prim,\ y} = A_{prim,\ y} \times BD_{prim,\ 2000} \times [1 + \delta(2000 - y)] \qquad \text{[Eq. 3]}$$
$$B_{man,\ y} = B_{tot,\ y} - B_{prim,\ y} \qquad \text{[Eq. 4]}$$
With $B_{prim,y}$, $B_{man,y}$ and $B_{tot,y}$ the biomass C stocks (MtC) in primary, managed and total forest in year y with y belonging to [1990-2020]; $A_{prim,y}$ the primary forest area in year y (ha); $BD_{prim,2000}$ the biomass C stock density (tC $ha^{-1}$) in primary forest in the year 2000 (as provided by Erb et al.[2]) and; δ the annual change in primary forest density derived from the literature survey (see Supplementary Tables 9-10).

## 3.2. Literature survey on Greening and browning of the arctic, boreal temperate, neotropical and paleotropical primary forests

We investigated the literature on greening and browning trends in arctic, boreal, temperate, paleotropical and neotropical regions in order to derive temporal trends in biomass density of primary forest in those forests. Using google scholar and web of science, we screened studies mentioning 'greening' OR 'browning' OR 'change in biomass density' AND 'primary forest' OR 'intact forest' OR 'wild forest' OR 'undisturbed forest' OR 'unmanaged forest' OR 'old-growth forest'.

### 3.2.1. Main findings

The data of Erb et al.[2] reveals that in 2000, 99% of the world's primary forests biomass was located in just 20 countries (the Russian Federation, Canada, USA, Brazil, Australia, Colombia, Venezuela, Peru, Bolivia, Suriname, Congo and DR Congo, Central African Rep., Guyana and French Guyana, Gabon, Ecuador, Indonesia and Malaysia). Of these, countries of the tropical zone and countries of the boreal zone (Canada and Russia) held respectively 43% and 47% of the total biomass of the world's primary forest biomass. This is consistent with data by Hubau et al.[25]; Tagesson et al.[1]; Potapov et al.[26].

In the case of tropical forests, the notion of primary forest as defined by FRA[5] is quite different from that of intact forest landscape (IFL) as defined by Potapov et al.[26], the latter often representing areas 5-7 times larger than the former, and comprising forests subject to some degree of direct human alteration such as small village exploitation and smallholder clearing[27,28].

By contrast to temperate and boreal primary forests where, with a few exceptions, only satellite data are available to quantify the decadal changes in biomass, several long-term series of direct biomass measurements in permanent sample plots provide useful indications about the trends in tropical above-ground biomass evolution[25,29,30]. These estimates can be compared with data from satellites observations.

### 3.2.2. Trends by macro-region

- **Neotropical region**

Phillips et al.[29] were the first to compile data from long-term monitoring plots of mature humid tropical forests from the middle of the 1970's to 1996. He found that in a majority of sites in the

humid Neotropical area (mostly Amazonian forest) gains by tree growth exceeded losses from death, leading to a net accumulation of c. 2 tDM/ha/yr from 1977 to 1981, 1 tDM/ha/yr in 1982-1986, 0.5 tDM/ha/yr in 1987-1991, 1 tDM/ha/yr in 1992-1996. Compared to an average biomass of 240 tDM/ha in this region (Erb et al.[2]), these variations represent respectively an increase of biomass density of 0.8, 0.4, 0.2, 0.4 % $yr^{-1}$. More recently, Brienen et al.[30] and Hubau et al.[25] showed that this net increasing trend in above-ground biomass density has declined by one third during the past decade compared to the 1990s. Using a combination of plot measurements and satellite data, Baccini et al.[11] estimated the net increase in biomass density of Amazonian primary forests from 2003 to 2014 to 0.67 % $yr^{-1}$.

- **Palaeotropical regions**

Phillips et al.[29] also provided estimates of biomass density changes for paleotropical forests (Africa, Asia and Australia), based however on less data than in America, showing a decreasing trend of -0.18 ± 0.59 tDM/yr (i.e. -0.04% $yr^{-1}$) from 1970 to 1996. This decreasing trend is confirmed by Hoshizaki et al.[31] based on data from the Pasoh Forest Reserve (Malaysia) showing a decrease of -0.45 tDM $ha^{-1}$ $yr^{-1}$ (-0.10 % $yr^{-1}$) from 1994 to 1998.

Concerning primary tropical rain forest in central Africa, several authors indicated a general long term trend of reduction of biomass density, explained by several factors among which a long-term drying trend[32], small village exploitation and small holder clearing[27,28,33], but also the disappearance of macro-mammals including elephants which regulates the forest's tree composition[34] and chimpanzee which disseminates the seeds of important species[35]. Zhou et al.[32], based on satellite data from several independent sensors estimate a loss of primary forest biomass density in the Congo basin by -0.03 %$yr^{-1}$ from 1988 to 2002.

Contrarily to these authors, Hubau et al.[25], based on the analysis of the data from 244 structurally intact African tropical forests, found that the carbon sink in live aboveground biomass has been stable from 1990-2015, at 0.66 tC $ha^{-1}$ $yr^{-1}$ (i.e. 0.3%/yr), in agreement with the figure reported by Lewis et al.[36] for the period 1968-2007.

- **Temperate and subtropical region**

For temperate forests, the data of Tan et al.[37] concerning the primary forest biomass density of the North of China, show an increase in carbon stock per ha of 0.4% $yr^{-1}$ during the period 1982 to 1999. This is confirmed by the data of Fang et al.[9] showing an increase of above-ground biomass density in Chinese so-called 'natural' forests by 0.46 % $yr^{-1}$ over the period 1970-2000. The data for Japan by the same authors suggests an increase rate as high as 1.3% $yr^{-1}$.

- **Boreal regions (Alaska, Canada, Russia and Scandinavia)**

Because, boreal and arctic forest are hardly accessible, almost all studies used methodological approach based on NDVI measurement from remote sensing data. Only the study by Ma et al.[38] is based on diachronic plot measurements from 96 sites of primary boreal forest, thus representing the most reliable study. In other studies, NDVI datasets are used as proxy for observing changes in terrestrial plant productivity and its response to climate. Satellite sensors measure solar radiation reflected by vegetation and NDVI is calculated from the red and near-infrared reflected light

channels. Whereas single NDVI measures serve as ‘‘snapshots’’ of forest cover, analysis of changes in NDVI over time can indicate changes in productivity and in biomass density. Nevertheless, NDVI are very poorly correlated with biomass density[39]. **Therefore, while we investigated (and hereafter summarized) the literature reporting NDVI trends in boreal forest (**Supplementary Table 9**), we only used the figures reported by Ma et al.**[38] **to derive trends in boreal primary forest density (**Supplementary Table **10).**

Only two studies investigated macro-regional trends in ‘undisturbed’ or ‘intact’ arctic, and boreal forests while one study investigated local trend in ‘undisturbed’ boreal forests in the North of the United States:

Sulla-Menashe et al.[40] estimated an **average trend in ΔNDVI over the 1984-2012 period of c. +0.0005-0.001 yr$^{-1}$ in undisturbed forests in Canada** (Fig. 2 of their article). They showed that the majority of changes in NDVI reflect disturbance and post-disturbance recovery processes and do not indicate widespread eco-physiological responses to climate change. NDVI values in undisturbed areas of Canada’s boreal forest were generally quite stable across the 28-year record that they examined. Undisturbed forests constituted 82% of the forested area in the panel data, of which 24% showed greening trends, 11% showed browning trends, 51% had no change (absolute value of the slope was less than 0.001 yr−1), and 13% showed absolute magnitudes of change greater than 0.001 yr−1, but which were statistically insignificant ($p$<0.05).

Similarly, Jin et al.[41] highlighted that 85.5% of boreal intact forest landscapes did not show a significant change in the NDVI from 2000 to 2013, and only 10.2% and 4.3% exhibited a statistically significant increase (greening) or decrease (browning) in NDVI, respectively. According to this study, 44.8% and 55.2% of these no-change intact forest landscapes were located in Eurasia (e.g., the East/West Siberian taiga) and North America (e.g., Eastern Canadian Shield taiga and Interior Alaska-Yukon lowland taiga), respectively. For the greening boreal intact forest landscapes, 47.1% and 52.9% were located in Eurasia and North America. For the browning intact forest landscapes, 27.9% and 72.1% of the IFLs were located in Eurasia and North America. These figures, together with the range in ΔNDVI shown in the spatial explicit map of Fig. 2 enables to estimate trend in ΔNDVI over the 2000-2013 of c. + 0.008 and 0.004 yr$^{-1}$ in Eurasia and North American boreal intact forest. This is consistent with the estimation found by Sulla-Menashe et al.[40] for North America.

Emmett et al.[42] assessed change in NDVI from 1989 to 2014 in the Greater Yellowstone Ecosystem (GYE), a site considered as one of the most intact ecosystems in the conterminous USA and which encompasses parts of four national forests and two national parks. Evergreen forests are the dominant forest in GYE and had a mean response of NDVI change of +0.001 yr$^{-1}$.

In addition, several studies investigated trends in boreal and temperate forests (including both primary and production forest). We report here after the main findings from these studies. Gao et al.[43] stressed out the existence of a ‘greening hiatus’ in **Eurasian boreal forest** in 1997. From 1982 to 1997, there were consistent and significant increases in vegetation productivity. After 1997, the increase in summer vegetation productivity stalled (i.e. the greening hiatus).

Although the figures displayed by Buermann et al.[44] of NDVI anomalies in the **Ural region** over the period 1989-2011 rather suggest an increase in NDVI over the entire period, their results also highlighted a “divergence” between warming and tree growth, with localized shifts to a negative relationship between temperature and growth, which is consistent with the ‘greening hiatus’ observed by Gao et al.[43]. These results highlighted that due to continued summer warming and in the absence of sustained increases in precipitation, a turning point has been reached around the mid- 1990’s that shifted western central Eurasian boreal forests into a warmer and drier regime. If such a regime shift would be sustained, the dieback of the boreal forest induced by heat and drought stress as predicted by vegetation models may proceed more rapidly than anticipated.

Parent and Verbyla[45] showed a declining trend in NDVI (2000-2009) in **Alaska boreal forest**, with the strongest browning trend occurring in eastern Alaska where the climate during the growing season is relatively dry and warm (significant slope spans from -0,005 to +0,01 $yr^{-1}$).

Nevertheless, it is worth noting that despite the observed browning trends in some part of the boreal forests, Alcarez-Segura et al.[46] emphasized that the presence of a bias in satellite datasets has broad implications for the evaluation of global and national carbon budgets. By comparing NDVI trend in North America with the GIMMS and the CCRS dataset, they could observe that the GIMMS dataset largely missed the postfire greening recovery, while the CCRS dataset, which has improved corrections and a much higher spatial resolution than GIMMS, did capture it under all conditions of fire severity. Positive NDVI trends in the Canadian boreal forest using the CCRS dataset were observed, thus contradicting the decline of vegetation activity observed based on the GIMMS dataset.

### 3.2.3. Summary of the temporal trends in primary forests of the world

**Supplementary Table S.** Summary of all the available data expressed in % of increase of biomass density $yr^{-1}$.

| Regions & countries | Authors | Period | Trend % yr-1 | Methods |
|---|---|---|---|---|
| Neotropical | | | | |
| Amazonian forest | Phillips et al.[29] | 1977-1981 | 0.83 | Plot measurements |
| | | 1982-1986 | 0.41 | Plot measurements |
| | | 1987-1991 | 0.21 | Plot measurements |
| | | 1992-1996 | 0.41 | Plot measurements |
| | Brienen et al.[30] | 2000-2010 | 0.03 | Plot measurements |
| | Hubau et al.[25] | 1980-1990 | 0.29 | Plot measurements |
| | | 1990-2000 | 0.44 | Plot measurements |
| | | 2000-2010 | 0.31 | Plot measurements |
| | | 2010-2015 | 0.20 | Plot measurements |
| | Baccini et al.[11] | 2003-2014 | 0.64 | combination of plot and satellite data |
| Paleotropical | | | | |
| Paleotropical | Phillips et al.[29] | 1970-1996 | -0.04 | Plot measurements |
| Malaysia (Pasoh Forest Reserve) | Hoshizaki et al.[31] | 1994-1998 | -0.10 | Plot measurements |
| Congo | Zhou et al.[32] | 2000-2012 | -0.126 | Satellite data (EVI Enhanced Vegetation Index) |

| | | | | |
|---|---|---|---|---|
| | | 1988-2002 | -0.03 | Satellite data (VOD vegetation optical depth) |
| African tropical forest | Hubau et al.[25] | 1980-1990 | 0.15 | Plot measurements |
| | | 1990-2000 | 0.31 | Plot measurements |
| | | 2000-2010 | 0.33 | Plot measurements |
| | | 2010-2015 | 0.31 | Plot measurements |
| African tropical forest | Lewis et al.[36] | 1968-2007 | 0.29 | Plot measurements |
| Boreal | | | | |
| All boreal area | Jin et al.[41] | 2000-2013 | 0.98 | Remote sensing + NDVI analysis |
| Eurasia | Jin et al.[41] | 2000-2013 | 1.33 | Remote sensing + NDVI analysis |
| Canada | Sulla-Manashe et al.[40] | 1984-2012 | 0.09-0.65 | Remote sensing (landsat) + NDVI analysis |
| Canada | Jin et al.[41] | 2000-2013 | 0.70 | Remote sensing + NDVI analysis |
| Canada | Ma et al.[38] | 1990-2000 | 0.28 | Diachronic measurements at 96 sites of boreal primary forest |
| Canada | Ma et al.[38] | 2000-2010 | -0.05 | Diachronic measurements at 96 sites of boreal primary forest |
| Northern USA | Emmett et al.[42] | 1989-2014 | 0.14 | Remote sensing + NDVI analysis |
| Temperate | | | | |
| China | Fang et al.[47] | 1986-1991 | -0.44 | Direct field measurements |
| | Fang et al.[47] | 1991-1996 | 1.15 | Direct field measurements |
| | Fang et al.[9] | 1985-1995 | 0.77 | Direct field measurements |
| | Fang et al.[9] | 1995-2005 | 0.31 | Direct field measurements |
| Southwestern Yunnan China | Tan et al.[37] | 2003-2006 | 2.85 | Eddy-covariance flux + biometric based methods |
| Japan | Fang et al.[9] | 1985-1995 | 0.98 | Direct field measurements |
| | Fang et al.[9] | 1995-2003 | 1.30 | Direct field measurements |

**Supplementary Table 10.** Summary of the temporal trend in biomass density of primary forests (values are provided in % of increase of biomass density per year)

| | Neotropical | Paleotropical | Boreal | Temperate |
|---|---|---|---|---|
| Average 1990-2000 | 0.37 | 0.24 | 0.28 | 0.97 |
| Average 2000-2020 | 0.33 | 0.33 | -0.05 | 0.19 |

## Supplementary References


1. Tagesson, T. *et al.* Recent divergence in the contributions of tropical and boreal forests to the terrestrial carbon sink. *Nat. Ecol. Evol.* **4**, 202–209 (2020).
2. Erb, K.-H. *et al.* Unexpectedly large impact of forest management and grazing on global vegetation biomass. *Nature* **553**, 73–76 (2018).
3. Li, W. *et al.* Gross and net land cover changes in the main plant functional types derived from the annual ESA CCI land cover maps (1992–2015). *Earth Syst. Sci. Data* **10**, 219–234 (2018).
4. Pan, Y. *et al.* A Large and Persistent Carbon Sink in the World's Forests. *Science* **333**, 988–993 (2011).
5. *Global Forest Resources Assessment 2020*. (FAO, 2020). doi:10.4060/ca9825en.
6. Magerl, A., Le Noë, J., Erb, K.-H., Bhan, M. & Gingrich, S. A comprehensive data-based assessment of forest ecosystem carbon stocks in the US 1907–2012. *Environ. Res. Lett.* **14**, 125015 (2019).
7. IGN. *L'Inventaire Forestier, Institut Géographique National*. https://inventaire-forestier.ign.fr/spip.php?rubrique226 (2018).
8. Bundesministerium Nachhaltigkeit und Tourismus. *Österreichische Waldinventur*. http://bfw.ac.at/rz/wi.home (2018).
9. Fang, J. *et al.* Forest biomass carbon sinks in East Asia, with special reference to the relative contributions of forest expansion and forest growth. *Glob. Change Biol.* **20**, 2019–2030 (2014).
10. Rajashekar, G. *et al.* Spatial distribution of forest biomass carbon (Above and below ground) in Indian forests. *Ecol. Indic.* **85**, 742–752 (2018).
11. Baccini, A. *et al.* Tropical forests are a net carbon source based on aboveground measurements of gain and loss. *Science* **358**, 230–234 (2017).
12. Achard, F. *et al.* Determination of tropical deforestation rates and related carbon losses from 1990 to 2010. *Glob. Change Biol.* **20**, 2540–2554 (2014).
13. Xu, L. *et al.* Spatial Distribution of Carbon Stored in Forests of the Democratic Republic of Congo. *Sci. Rep.* **7**, 15030 (2017).
14. Cannell, M.G.R. *World forest biomass and primary production data*. (Academic Press, 1982).
15. Luyssaert, S. *et al.* $CO_2$ balance of boreal, temperate, and tropical forests derived from a global database. *Glob. Change Biol.* **13**, 2509–2537 (2007).
16. Anderson-Teixeira, K. J., Wang, M. M. H., McGarvey, J. C. & LeBauer, D. S. Carbon dynamics of mature and regrowth tropical forests derived from a pantropical database (TropForC-db). *Glob. Change Biol.* **22**, 1690–1709 (2016).
17. Hoelzemann, J. J. Global Wildland Fire Emission Model (GWEM): Evaluating the use of global area burnt satellite data. *J. Geophys. Res.* **109**, D14S04 (2004).
18. Mouillot, F., Narasimha, A., Balkanski, Y., Lamarque, J.-F. & Field, C. B. Global carbon emissions from biomass burning in the 20th century: GLOBAL CARBON EMISSIONS FROM BIOMASS BURNING. *Geophys. Res. Lett.* **33**, n/a-n/a (2006).
19. Yang, J. *et al.* A growing importance of large fires in conterminous United States during 1984–2012. *J. Geophys. Res. Biogeosciences* **120**, 2625–2640 (2015).
20. Kloster, S. *et al.* Fire dynamics during the 20th century simulated by the Community Land Model. *Biogeosciences* **7**, 1877–1902 (2010).

21. van Leeuwen, T. T. *et al.* Biomass burning fuel consumption rates: a field measurement database. *Biogeosciences* **11**, 7305–7329 (2014).
22. Ito, A. Global estimates of biomass burning emissions based on satellite imagery for the year 2000. *J. Geophys. Res.* **109**, D14S05 (2004).
23. Van Der Werf, G. R., Randerson, J. T., Collatz, G. J. & Giglio, L. Carbon emissions from fires in tropical and subtropical ecosystems: CARBON EMISSIONS FROM TROPICAL FIRES. *Glob. Change Biol.* **9**, 547–562 (2003).
24. Luyssaert, S. *et al.* Old-growth forests as global carbon sinks. *Nature* **455**, 213–215 (2008).
25. Hubau, W. *et al.* Asynchronous carbon sink saturation in African and Amazonian tropical forests. *Nature* **579**, 80–87 (2020).
26. Potapov, P. *et al.* The last frontiers of wilderness: Tracking loss of intact forest landscapes from 2000 to 2013. *Sci. Adv.* **3**, e1600821 (2017).
27. Zhuravleva, I. *et al.* Satellite-based primary forest degradation assessment in the Democratic Republic of the Congo, 2000–2010. *Environ. Res. Lett.* **8**, 024034 (2013).
28. Tyukavina, A. *et al.* Congo Basin forest loss dominated by increasing smallholder clearing. *Sci. Adv.* **4**, eaat2993 (2018).
29. Phillips, O. L. Changes in the Carbon Balance of Tropical Forests: Evidence from Long-Term Plots. *Science* **282**, 439–442 (1998).
30. Brienen, R. J. W. *et al.* Long-term decline of the Amazon carbon sink. *Nature* **519**, 344–348 (2015).
31. Hoshizaki, K. *et al.* Temporal and spatial variation of forest biomass in relation to stand dynamics in a mature, lowland tropical rainforest, Malaysia: Biomass variation in a tropical forest. *Ecol. Res.* **19**, 357–363 (2004).
32. Zhou, L. *et al.* Widespread decline of Congo rainforest greenness in the past decade. *Nature* **509**, 86–90 (2014).
33. Tyukavina, A. *et al.* Aboveground carbon loss in natural and managed tropical forests from 2000 to 2012. *Environ. Res. Lett.* **10**, 074002 (2015).
34. Berzaghi, F. *et al.* Carbon stocks in central African forests enhanced by elephant disturbance. *Nat. Geosci.* **12**, 725–729 (2019).
35. Giresse, P., Maley, J. & Chepstow-Lusty, A. Understanding the 2500 yr BP rainforest crisis in West and Central Africa in the framework of the Late Holocene: Pluridisciplinary analysis and multi-archive reconstruction. *Glob. Planet. Change* **192**, 103257 (2020).
36. Lewis, S. L. *et al.* Increasing carbon storage in intact African tropical forests. *Nature* **457**, 1003–1006 (2009).
37. Tan, Z. *et al.* Carbon balance of a primary tropical seasonal rain forest. *J. Geophys. Res.* **115**, D00H26 (2010).
38. Ma, Z. *et al.* Regional drought-induced reduction in the biomass carbon sink of Canada's boreal forests. *Proc. Natl. Acad. Sci.* **109**, 2423–2427 (2012).
39. Tan, K., Piao, S., Peng, C. & Fang, J. Satellite-based estimation of biomass carbon stocks for northeast China's forests between 1982 and 1999. *For. Ecol. Manag.* **240**, 114–121 (2007).
40. Sulla-Menashe, D., Woodcock, C. E. & Friedl, M. A. Canadian boreal forest greening and browning trends: an analysis of biogeographic patterns and the relative roles of disturbance versus climate drivers. *Environ. Res. Lett.* **13**, 014007 (2018).

41. Jin, J., Wang, Y., Jiang, H. & Cheng, M. Recent NDVI-Based Variation in Growth of Boreal Intact Forest Landscapes and Its Correlation with Climatic Variables. *Sustainability* **8**, 326 (2016).
42. Emmett, K. D., Renwick, K. M. & Poulter, B. Disentangling Climate and Disturbance Effects on Regional Vegetation Greening Trends. *Ecosystems* **22**, 873–891 (2019).
43. Gao, X., Liang, S. & Sauer, J. Greening Hiatus in Eurasian Boreal Forests Since 1997 Caused by a Wetting and Cooling Summer Climate. *J. Geophys. Res. Biogeosciences* **125**, (2020).
44. Buermann, W. *et al.* Recent shift in Eurasian boreal forest greening response may be associated with warmer and drier summers: BUERMANN ET. AL.; BOREAL FORESTS UNDER CLIMATE CHANGE. *Geophys. Res. Lett.* **41**, 1995–2002 (2014).
45. Parent, M. B. & Verbyla, D. The Browning of Alaska's Boreal Forest. *Remote Sens.* **2**, 2729–2747 (2010).
46. Alcaraz-Segura, D., Chuvieco, E., Epstein, H. E., Kasischke, E. S. & Trishchenko, A. Debating the greening vs. browning of the North American boreal forest: differences between satellite datasets. *Glob. Change Biol.* **16**, 760–770 (2010).
47. Fang, J. Changes in Forest Biomass Carbon Storage in China Between 1949 and 1998. *Science* **292**, 2320–2322 (2001).
